# Staged stenting strategy of acutely wide-neck ruptured intracranial aneurysms: A meta-analysis and systematic review

**DOI:** 10.3389/fneur.2023.1070847

**Published:** 2023-02-03

**Authors:** Yanpeng Wei, Xiaoxi Zhang, Renkun Zhang, Guanghao Zhang, Chenghao Shang, Rundong Chen, Dan Li, Meihua Huyan, Congyan Wu, Kang Zong, Zhengzhe Feng, Dongwei Dai, Qiang Li, Qinghai Huang, Yi Xu, Pengfei Yang, Rui Zhao, Qiao Zuo, Jianmin Liu

**Affiliations:** Neurovascular Center, Changhai Hospital, Naval Medical University, Shanghai, China

**Keywords:** wide-neck, ruptured intracranial aneurysms (RIA), staged stenting, complications, initial coiling

## Abstract

**Objective:**

In the study, we explored the safety and effectiveness of staged stenting strategy for acutely wide-neck ruptured intracranial aneurysms.

**Methods:**

Online databases, including PubMed, EMBASE, the Cochrane database, and Web of Science, were retrospectively and systematically searched. The main observation indicators were the procedure-related complication rate, complete occlusion rate, and favorable clinical outcome. Meta-analysis was performed using a random or fixed effect model based on heterogeneity.

**Results:**

A total of 5 studies with 143 patients were included. The hemorrhagic complication rate of the initial coiling and staged stenting was 2.8% (4 of 143) and 0, respectively. The ischemic complication rate of the coiling and supplemental stenting was 3.5% (5 of 143) and 2.9% (4 of 139), respectively. There were no deaths due to procedure-related complications in two stages. The aneurysm complete occlusion rate was 25% (95% CI, 0.13–0.03; I^2^ = 4.4%; *P* = 0.168) after initial coiling, 54% (95% CI, 0.63–0.64; I^2^ = 0%; *P* = 0.872) after staged stenting, and 74% (95% CI, 0.66–0.81; I^2^ = 56.4%; *P* = 0.562) at follow-up, respectively. Favorable clinical outcome rate 74% (95% CI, 0.61–0.86; I^2^ = 50.5%; *P* = 0.133) after discharge of initial coiling treatment, and 86% (95% CI, 0.80–0.92; I^2^ = 0; *P* = 0.410) after discharge from stenting, and 97% (95% CI, 0.93–1.01; I^2^ = 43.8%; *P* = 0.130) at follow-up.

**Conclusion:**

Staged stenting treatment of wide-neck RIA with coiling in the acute phase followed by delayed regular stent or flow-diverter stent had high aneurysm occlusion rate, favorable clinical outcome rate and low procedure-related complication rate. A more dedicated and well-designed controlled study is warranted for further evaluation of staged stenting treatment compared to SCA in wide-neck RIA.

## Introduction

Endovascular embolization has become the standard treatment strategy for ruptured intracranial aneurysms (RIA) (1). The indications of endovascular embolization are increasingly well defined, however it remains controversial for the wide-neck RIA (2). For wide-neck RIA, stents are often necessary to provide permanent protection for the coil inside the aneurysm sac, which may prevent coil prolapse or migration during the procedure (3). Stent assisted coiling (SAC) also allows to obtain a better immediate occlusion, however, the application of stent and antiplatelet medication can lead to more unexpected ischemic and hemorrhagic complications in the acute phase of subarachnoid hemorrhage (SAH) (4, 5). The overall rate of perioperative complications of SAC in RIA was about 20.2% as we previously reported, which was significantly higher than coiling only (6).

To avoid the high complication risk of SAC due to antiplatelet medication use in acute phase of SAH for wide-neck RIA, staged stenting strategy was gradually being applied. In a recent study, acute coiling followed by staged stenting in the treatment of 20 RIA showed that the perioperative complication during acute coiling was 3.7% and the staged treatment was no complication occurred, which suggested that staged stenting could avoid the potential risk of stent placement in the acute phase of SAH (7). As reported, staged stenting strategy was divided into two stages (7–11). The patients underwent conventional coiling in the acute phase of SAH, the purpose of which was to embolize the target aneurysms to avoid early rebleeding without stenting. Then adequate antiplatelet medication and supplemental stenting treatment (regular stent or flow-diverter stent) were scheduled after 2 weeks at least when the patient's condition was stable. Considering the distinctive characteristics of wide-neck RIA, the argument of whether staged stenting strategy would be more appropriate for managing wide-neck RIA remains unclear. Therefore, we performed a meta-analysis to evaluate the safety and outcomes of staged stenting treatment for wide-neck RIA.

## Materials and methods

### Literature search strategy

This systematic review and single-arm meta-analysis was conducted in accordance with the PRISMA guidelines (12). A systematic search and critical review of the reported data were from January 2006 to June 2022. A thorough search of published English language literature was performed using PubMed, EMBASE, the Cochrane database, and Web of Science. The terms “ruptured”, “intracranial aneurysms”, “cerebral aneurysms”, “intracranial aneurysm”, “cerebral aneurysm”, “staged”, “subtotal”, “planned”, “partial”, “targeted”, “stent”, and “flow-diverter” were combined as either keywords or Medical Subject Headings terms to identify all eligible studies. The reference lists of included studies were searched manually. All identified articles were systematically evaluated using the inclusion and exclusion criteria.

### Selection criteria

The inclusion criteria were: (1) studies reported patients with wide-neck RIA verified by CT scan and CTA /MRA/DSA, who underwent staged stenting (regular stent or flow-diverter stent) treatment; (2) studies included at least 10 patients; (3) studies reported the clinical or angiographic outcomes of aneurysms. The exclusion criteria were as follows: (1) unextractable or unclear data; (2) second staged treatment using alternative modalities; (3) duplicated reports; (4) fusiform, dissecting, mycotic aneurysms; (5) unpublished studies, reviews, meta-analyses, comments, letters, pilot studies, conference-only reports, technical notes, case reports, and abstract only and non-English language studies. The database search and study selection were performed by two junior physicians (Wei and Zhang) independently, with disagreements settled by the senior physicians (Zuo and Liu).

### Data extraction and item definition

The following information was extracted for the included studies: author, country, publication year, number of patients, baseline patient information, time between coiling and stent, complications, and so forth. The investigators were contacted if additional data were necessary.

The main observation indicators include the following: (1) Perioperative procedure-related complications and mortality in both phases. Procedure-related complications included hemorrhagic and ischemic complications. Hemorrhagic complications were defined as intraoperative aneurysm rupture and early rebleeding in two stages. Ischemic complications included acute in-stent thrombus formation, thromboembolic event, parent or branch artery occlusion. Procedure-related mortality was defined as death caused by a procedure-related complication other than deterioration of a severe condition. (2) The intracranial aneurysms complete occlusion rate immediately after both procedures and at follow-up. The intracranial aneurysm occlusion was evaluated using Raymond-Roy grade scale: (I) complete occlusion, (II) neck remnant, and (III) incomplete occlusion (13). (3) Favorable clinical outcome after both procedures immediately and at follow-up. Favorable clinical outcomes were defined as a modified Rankin scale score of 0–2.

### Quality assessment and statistical analysis

Assessment of study quality was performed using a Joanna Briggs institute scale for 6 studies (Table 1).

**Table 1:** Joanna Briggs institute scale.

**Parameter**	**Feng et al. (8)**	**Benjamin et al. (10)**	**Brinjikji et al. (9)**	**Mehm et al. (7)**	**Howard et al. (11)**
1. Clear criteria for inclusion	√	√	√	√	√
2. Condition measured in a standard, reliable way	√	√	√	√	√
3. Valid methods used for identification of the condition	√	√	√	√	√
4. Consecutive inclusion of participants	√	※	√	※	※
5. Complete inclusion of participants	√	※	√	※	※
6. Clear reporting of the demographics	√	√	√	√	√
7. Clear reporting of clinical information	√	√	√	√	√
8. Clear reporting outcomes or follow up results	√	√	√	√	※
9. Clear reporting of the presenting site(s)/clinic(s)	√	√	√	√	√
10. Appropriate statistical analysis	√	√	√	√	√

Studies with 6 positive answers were defined as good quality.

A meta-analysis was performed using Stata, version 16 (Stata Corp., College Station, Texas, USA). Continuous variables are presented as mean values or median and range. Dichotomous variables are presented as risk ratios with 95% confidence intervals (CI). Statistical heterogeneity was assessed using I^2^, a random effect model was used for analysis if the I^2^ was >50%, and a sensitivity analysis was further performed. For analysis with an I^2^ <50%, a fixed-effect model was used and a sensitivity analysis was not performed. Significance was set at *P* < 0.05.

## Results

### Literature search, study characteristics, and quality assessment

**Figure 1:**
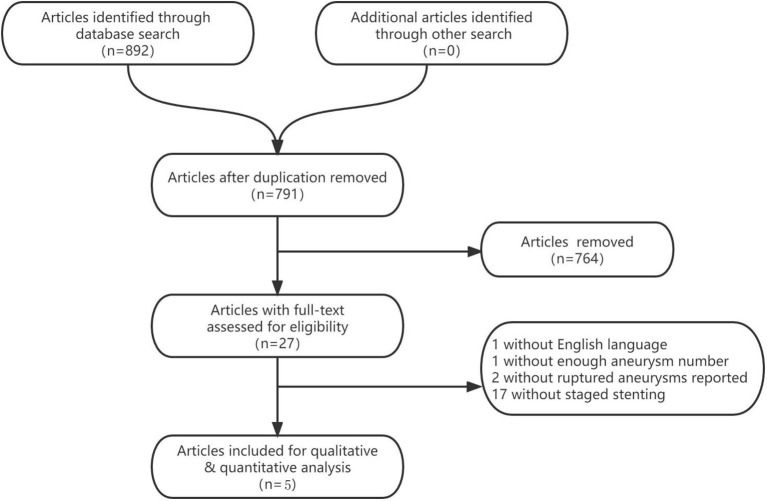
Flowchart of study selection.

The literature search process was presented in a Preferred Reporting Items for flow chart showing the number of studies screened and excluded at each stage (Figure 1). The basic characteristics of the included eligible studies were summarized in Table 2. All included studies were retrospective studies using data from retrospective or prospective databases. The quality of the included studies using a Joanna Briggs institute scale (score range, 0~10), with 6 positive answers taken to define a good quality study (Table 1).

**Table 2:** The basic characteristics of the included eligible studies.

**Investigator**	**Study period**	**Country**	**Patient number in initial coiling**	**Age (years)**	**Female sex *n* (%)**	**Anterior circulation *n* (%)**	**Aneurysm size (mm)**	**Neck size (mm)**	**Patient number in staged stenting**	**Time between coiling and stent (days)**	**Follow-up month (Angiographic)**	**Moderate clinical status^⋆^**
Feng et al. (8)	2006/11–2016/9	China	47	55.4 ± 11.6	28 (59.57%)	46 (98%)	4.9 ± 4.0	4.2 ± 2.9	47	29.4	6.7~36.4	38.30%
Benjamin et al. (10)	2012/1–2017/6	Belgium	23	50	15 (65.2%)	21 (91%)	7.1	3.4	23	24.3	6	82.6%^⋆^
Brinjikji et al. (9)	2009/4–2014/8	Italy	31	52.1 ± 11.1	17 (55%)	25 (84%)	15.8 ± 7.9	NA	27	119 (8–700)^Δ^	19.2 ± 12.0	64.50%
Mehm et al. (7)	2016/9–2020/12	Turkey	20	51.6 ± 14.2^✰^	49 (45%)^✰^	17 (85%)	8.97 ± 3.57	4.29 ± 0.85	20	87.71 ± 36.89	6	55%
Howard et al. (11)	NA	USA	22	57 (36–83)^Δ^	19 (86%)	21 (95%)	8.8 (2.3–24)^Δ^	4.7 (1.8–8.3)^Δ^	22	24.5 (3.5–105) ^Δ^	6.0 (1.8–8.3)^Δ^	23.80%

Data presented as mean or mean ± standard deviation or *n* (%), unless otherwise stated; ^✰^, The description includes other groups; NA, not reported; ^Δ^, median (range); Moderate clinical status. ^⋆^, Hunt-Hess(1–2)/WFSN(1–2)/GCS(9–15).

We identified 892 studies through the database search, and a total of 5 studies (7–11) were included in the present meta-analysis, with 143 ruptured intracranial aneurysms patients (Table 2).

### Analysis of safety and outcomes

In all the 5 studies, the patients underwent conventional coiling in the acute phase of SAH without antiplatelet therapy. In 3 studies, the researchers described detailed antiplatelet strategy when patients underwent the supplemental stenting treatment. The regular dual antiplatelet drugs were administered for at least 3 days before stenting or a loading dose of dual antiplatelet drugs was used before the procedure.

The hemorrhagic complications only occurred in 4 patients (4 of 143, 2.8%) in perioperative of coiling. Among them the intraoperative aneurysm bleeding rate was 2.1% (3/143), and the early rebleeding rate was 0.7% (1 of 143). The ischemic complications occurred in 5 patients (5 of 139, 3.5%) in perioperative of coiling and 4 patients (4 of 139, 2.8%) in perioperative of stenting. There were no deaths due to procedure-related complications in two stages (Table 3).

**Table 3:** Perioperative procedure-related complications and mortality in both stages.

**Investigator**	**Perioperative complication of initial coiling (n)**				**Perioperative complication of staged stenting (n)**			

	**Patients number**	**Hemorrhagic**	**Ischemic**	**Mortality**	**Patients number**	**Hemorrhagic**	**Ischemic**	**Mortality**
Feng et al. (8)	47	0	0	0	47	0	0	0
Benjamin et al. (10)	23	0	0	0	23	0	1	0
Brinjikji et al. (9)	31	3	4	0	27	0	2	0
Mehm et al. (7)	20	1	0	0	20	0	0	0
Howard et al. (11)	22	0	1	0	22	0	1	0
Total	143	4	5	0	139	0	4	0

**Figure 2:**
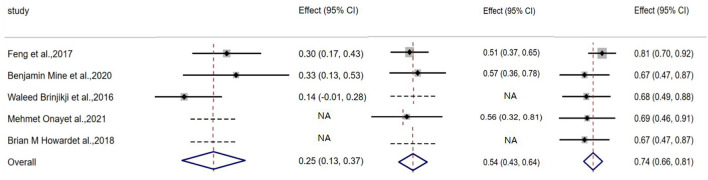
Plot showing complete occlusion rate after coiling, stenting and at follow up.

The aneurysm complete occlusion rate was 25% (95% CI, 0.13–0.03; I^2^ = 4.4%; *P* = 0.168) after initial coiling, 54% (95% CI, 0.63–0.64; I^2^ = 0%; *P* = 0.872) after staged stenting, and 74% (95% CI, 0.66–0.81; I^2 =^ 56.4%; *P* = 0.562) at follow-up, respectively (Table 4, Figure 2).

**Table 4:** The aneurysm complete occlusion rate immediately after both stages and at follow-up.

**Investigator**	**Immediate outcomes** **after coiling**			**Complete occlusion rate**	**Immediate outcomes after stenting**			**Complete occlusion rate (%)**	**Outcomes at follow up**			**Complete occlusion rate**

	**I**	**II**	**III**		**I**	**II**	**III**		**I**	**II**	**III**	
Feng et al. (8)	14	22	11	30%	24	18	5	51%	38	6	3	81.0%
Benjamin et al. (10)	7	13	3	30%	12	11	0	52%	14	6	1	67.0%
Brinjikji et al. (9)	3	13	15	10%			NA		15	3	4	68.0%
Mehm et al. (7)	0	0	20	0%	9	5	6	45%	11	4	1	68.0%
Howard et al. (11)	0	2	20	0%			NA		14	3	4	67.0%

**Figure 3:**
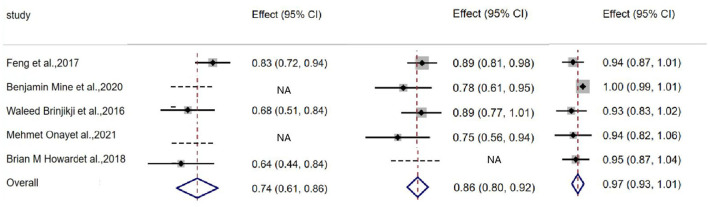
Plot showing favorable clinical outcome after coiling, stenting and at follow up.

Favorable clinical outcome rate was 74% (95% CI, 0.61–0.86; I^2^ = 50.5%; *P* = 0.133) after discharge of initial coiling treatment, and 86% (95% CI, 0.80–0.92; I^2^ = 0; *P* = 0.410) after discharge from stenting, and 97% (95% CI, 0.93–1.01; I^2^ = 43.8%; *P* = 0.130) at follow-up, respectively (Table 5, Figure 3).

**Table 5:** Favorable clinical outcome immediately after both procedures and at follow-up.

**Investigator**	**Favorable clinical outcomes (*****n*** **%)**		
	**After discharge from coiling**	**After discharge from stenting**	**Follow-up**
Feng et al. (8)	83.0%	89.3%	93.6%
Benjamin et al. (10)	NA	78.2%	100.0%
Brinjikji et al. (9)	67.7%	88.9%	92.6%
Mehm et al. (7)	NA	75.0%	93.8%
Howard et al. (11)	63.6%	NA	95.4%

### Sensitivity analysis and publication bias

**Figure 4:**
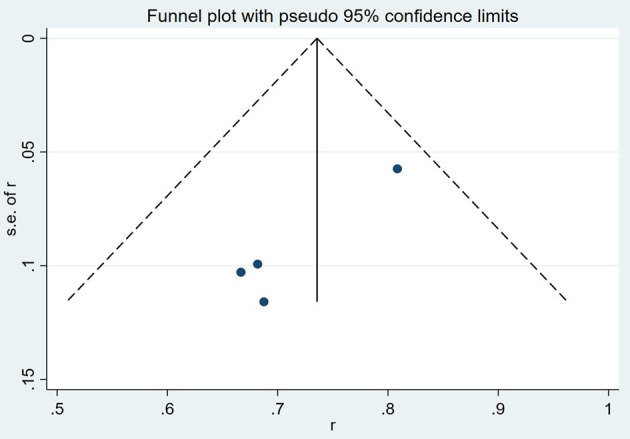
Funnel plot analysis of complete occlusion rate at follow up.

**Figure 5:**
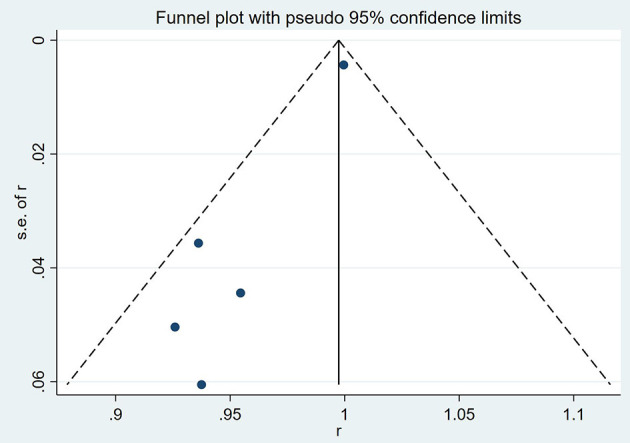
Funnel plot analysis of favorable clinical outcome at follow up.

The results of a funnel plot analysis of favorable clinical outcome at discharge was shown in Figures 4, 5, which indicated obvious publication bias. Similar results were also obtained for the immediate occlusion rate (Figures 4, 5).

## Discussion

Endovascular treatment has long been part of the standard strategies in the treatment of RIA to prevent aneurysm rebleeding after ISAT trial (14). With the development of skills and devices, the use of intracranial stents for endovascular treatment of intracranial aneurysms has dramatically widened its indications to wide-neck RIA (15, 16). However, SAC is not the first option for treating wide-neck RIA in the acute phase of SAH. Studies related to SAC of RIA have been performed in several centers, and the use of intracranial stents may be associated with a higher risk of ischemic and hemorrhagic complications if the antiplatelet therapy is not proper (4, 17). In our previous study on SAC, the ischemic complication rate was 3.1–8.1% and the hemorrhagic complication rate was 4.5–6.2% in different periods and with different stents (3, 18). Meanwhile, antiplatelet therapy may also increase the risk of hemorrhagic complications during invasive surgeries that may be necessary within the early time of severe SAH such as ventricular drainage, hematoma clearance, or decompressive craniectomy. The study of Kung et al. (19) showed that the surgery-related bleeding risk was approximately three times higher in patients who used dual antiplatelet drugs with RIA than in those without dual antiplatelet drugs use (95% CI 1.46–8.04, *p* = 0.0048).

To effectively reduce the high rate of hemorrhagic and ischemic complications, several studies have reported the staged treatment of wide-neck RIA with coiling in the acute phase followed by delayed stenting, which may be a safe and effective strategy compared to SAC for acutely ruptured aneurysms. It allowed patients to avoid dual antiplatelet therapy during the acute phase and allowed for transitioning of the patients to a more subacute phase when the patient and aneurysm were stabilized, and dual antiplatelet therapy was safer.

Waldau et al. (20) firstly described the staged stent strategy for wide-neck RIA in 2010, with 5 patients who had complex ruptured aneurysms receiving intentional partial coiling dome protection and staged stent placement, and none of them experienced aneurysm rerupture before the supplementary stent treatment. We previously reported a series of 47 RIA patients with staged stenting treatment, and the results showed that no perioperative procedure-related complications and related death in both phases (8). Moreover, Onay et al. (7) proposed a different and radical technique of only targeted aneurysm bleb embolization and staged stenting treatment. They used small coils to embolize the bleb only instead of embolizing the aneurysm sac, which may lead to an increase in the formation of thromboses at the bleb and ensure the stabilization of the bleb to avoid early rebleeding as they considered. In their study, although the early occlusion rate was 0, no patient suffered rebleeding before the second-stage stenting and the occlusion rate was 68% at follow-up. The safety of this new approach needs to be further validated with more studies.

Among the 143 included patients in this meta-analysis, only 4 (2.8%) patients had hemorrhagic events, and 5 (3.5%) patients had ischemic events, with no deaths due to procedure-related complications. The rate of complications seemed lower than our previous study about SAC (3, 18). Also in Mehmet's study, the staged stenting strategy had significantly lower complication rates compared to the SAC (*p* = 0.047).

In the previous meta-analysis of wide-neck RIA, the immediate occlusion rate of single coiling was 64.2% (6). It was indeed an interesting result that a low rebleeding rate was observed after the staged stenting strategy even with a relatively low rate of aneurysm immediate occlusion (25%). We shared the view of these researchers that non-dense embolization of wide-neck RIA may provide adequate protection against aneurysms rerupture in the acute phase. More evidence is still needed on whether non-dense embolization could prevent aneurysm rerupture.

At the same time, compared with our previous study about SAC of wide-neck RIA, the results of this meta-analysis showed similar rate of favorable clinical outcome (94 vs. 85.6%) and long-term angiographic complete occlusion (75 vs. 74%) at follow-up (3). Thus, staged stenting strategy may be a safe and effective way for wide-neck RIA treatment without antiplatelet management in the acute phase and with adequate antiplatelet preparation before the second-stage stent placement.

There were some limitations in the meta-analysis. First, in these 5 searched publications, the sample size was small. And only in 1 study the results of the staged stenting strategy were compared with SAC in acute phase. Therefore, we only performed a single-arm meta-analysis to summarize the preliminary results and experiences about the staged stenting strategy for wide-neck RIA. Second, the enrolled patients were carefully selected and treated with staged stenting strategy. These results may not truly represent the safety and effectiveness of staged stenting strategy. That was an unavoidable drawback of retrospective studies.

Third, the antiplatelet strategies differed across these studies, which may affect complications when supplementing stents. A more dedicated and well-designed controlled study is warranted for further evaluation of staged treatment with stent compared to SAC in acute phase of wide-neck RIA.

## Conclusion

Staged stenting strategy of wide-neck RIA with coiling in the acute phase followed by delayed regular stent or flow-diverter stent had low procedure-related complication rates, favorable clinical outcome and high aneurysms occlusion rate at follow-up based on this single arm meta-analysis. We advocate for future prospective, randomized controlled trials of this promising therapy.

## Data availability statement

The raw data supporting the conclusions of this article will be made available by the authors, without undue reservation.

## Author contributions

YW, XZ, and RZhan made substantial contributions to the conception and design, acquisition of data, analysis, and drafting of the manuscript. GZ, CS, RC, DL, MH, CW, and KZ searched for relevant studies and selected the studies. ZF, DD, QL, QH, YX, PY, RZhao, QZ, and JL assisted in the evaluation of analysis and their interpretation. All authors read and approved the final manuscript.
